# The use of polygenic risk scores in pre-implantation genetic testing: an unproven, unethical practice

**DOI:** 10.1038/s41431-021-01000-x

**Published:** 2021-12-17

**Authors:** Francesca Forzano, Olga Antonova, Angus Clarke, Guido de Wert, Sabine Hentze, Yalda Jamshidi, Yves Moreau, Markus Perola, Inga Prokopenko, Andrew Read, Alexandre Reymond, Vigdis Stefansdottir, Carla van El, Maurizio Genuardi, on behalf of the Executive Committee of the European Society of Human Genetics, Borut Peterlin, Carla Oliveira, Karin Writzl, Gunnar Douzgos Houge, the Public and Professional Policy Committee of the European Society of Human Genetics, Christophe Cordier, Heidi Howard, Milan Macek, Béla Melegh, Alvaro Mendes, Dragica Radojkovic, Emmanuelle Rial-Sebbag, Fiona Ulph, Yalda Jamshidi

**Affiliations:** 1 Clinical Genetics Department, Guy’s and St Thomas NHS Foundation Trust, London, UK; 2 Department of Medical Genetics, Medical University of Sofia, Sofia, Bulgaria; 3 Division of Cancer and Genetics, School of Medicine, Cardiff University, Cardiff, Wales UK; 4 Maastricht University, Maastricht, The Netherlands; 5 Human Genetics, Heidelberg, Germany; 6 Genetics Research Centre, Molecular and Clinical Sciences Institute, St George’s University of London, London, UK; 7 University of Leuven ESAT-STADIUS, B-3001 Leuven, Belgium; 8 Finnish Institute for Health and Welfare (THL), Biomedicum 1, Haartmaninkatu 8, 00290 Helsinki, Finland; 9 Department of Clinical & Experimental Medicine, University of Surrey, Guildford, UK; 10 UMR 8199 - EGID, Institut Pasteur de Lille, CNRS, University of Lille, F-59000 Lille, France; 11 Institute of Biochemistry and Genetics, Ufa Federal Research Centre, Russian Academy of Sciences, Ufa, Russian Federation; 12 Centre for Genomic Medicine, St Mary’s Hospital, Manchester, M13 0JH England; 13 Center for Integrative Genomics, University of Lausanne, CH-1015 Lausanne, Switzerland; 14 Department of Genetics and Molecular Medicine, Landspitali University Hospital, Reykjavik, Iceland; 15 Section Community Genetics, Department of Clinical Genetics and Amsterdam Public Health Research Institute, Amsterdam UMC, Vrije Universiteit Amsterdam, Amsterdam, The Netherlands; 16 UOC Genetica Medica, Dipartimento di Scienze di Laboratorio e Infettivologiche, Fondazione Policlinico Universitario A. Gemelli IRCCS, Rome, Italy; 17 Sezione di Medicina Genomica, Dipartimento di Scienze della Vita e Sanità Pubblica, Università Cattolica del Sacro Cuore, Rome, Italy; 18 Clinical Institute for Genomic Medicine, University Medical Center Ljubljana, Ljubljana, Slovenia; 19 Department of Pathology, Faculty of Medicine, University of Porto, PT, Porto, Portugal; 20 Instituto de Investigação e Inovação em Saúde (i3S), University of Porto, PT, Porto, Portugal; 21 Institute of Molecular Pathology and Immunology of the University of Porto (Ipatimup), University of Porto, PT, Porto, Portugal; 22 Department of Medical Genetics, Haukeland University Hospital, 5021 Bergen, Norway; 23 Department of Genetics, SYNLAB Suisse SA, Chemin d’Entre Bois 21, 1018 Lausanne, Switzerland; 24 Researcher Medical Ethics, Lund University, Uppsala, Sweden; 25 Chalmers University (part of GENIE initiative), Uppsala, Sweden; 26 Department of Biology and Medical Genetics, 2nd Faculty of Medicine, Charles University and Motol University Hospital, V Uvalu 84, Prague, CZ15006 Czech Republic; 27 Department of Medical Genetics, University of Pécs, Szigeti 12., H-7624 Pécs, Hungary; 28 UnIGENe and Centre for Predictive and Preventive Genetics, IBMC—Institute for Molecular and Cell Biology, i3S—Instituto de Investigação e Inovação em Saúde, Universidade do Porto, Porto, Portugal; 29 Laboratory for Molecular Biology, Institute of Molecular Genetics and Genetic Engineering, University of Belgrade, Belgrade, Serbia; 30 CERPOP, UMR 1295, Inserm, CERPOP, UMR 1295, Inserm, Université de Toulouse-Université Paul Sabatier-Toulouse III, Responsable Equipe BIOETHICS: Trajectoires d’innovations en santé:enjeux bioéthiques et sociétaux, Toulouse, France; 31 Plateforme Sociétale “Génétique et Société, GIS Genotoul, Génopole Toulouse Midi-Pyrénées, 37, allées Jules Guesde, 31073 Toulouse Cedex, France; 32 Manchester Centre of Health Psychology, Division of Psychology and Mental Health, School of Health Sciences, Manchester Academic Health Science Centre, University of Manchester, Coupland Street, Manchester, M13 9PL England

**Keywords:** Medical ethics, Disease prevention

## Abstract

Polygenic risk score analyses on embryos (PGT-P) are being marketed by some private testing companies to parents using in vitro fertilisation as being useful in selecting the embryos that carry the least risk of disease in later life. It appears that at least one child has been born after such a procedure. But the utility of a PRS in this respect is severely limited, and to date, no clinical research has been performed to assess its diagnostic effectiveness in embryos. Patients need to be properly informed on the limitations of this use of PRSs, and a societal debate, focused on what would be considered acceptable with regard to the selection of individual traits, should take place before any further implementation of the technique in this population.

## Introduction

Polygenic risk scores (PRSs) are estimates of an individual’s susceptibility to a specific complex trait obtained by aggregating the effects of dozens, thousands, and potentially millions of genetic variants associated with that specific trait into a single figure. Some private companies have begun to market PRS analyses on embryos to prospective parents through the use of in vitro fertilisation and pre-implantation genetic testing (PGT; PGT-P) [1–4]

This practice raises many concerns.

Complex traits are determined by a combination of genes and environment, and PRSs can only capture a part of the genetic component – that which is derived from the cumulative effects of many genetic variants of small individual effect. PRSs themselves should be calculated using their effects from the ethnic group the parents belong to. The estimation of PRSs for children of parents from diverse ethnic origins is not yet possible to determine correctly. For risks to be calculated as accurately as possible, PRSs should be combined with the effects of non-genetic factors from an individual’s life history such as environment, nutrition, and physical activity. Furthermore, the effects of the genetic factors may interact with each other as well as with changes in lifestyle and clinical risk factors throughout an individual’s life, and these interactions may be difficult to account for when calculating the PRS. The concomitant occurrence of rare genetic variants of major effect, whose presence might be unknown, can influence hugely the calculation of the PRS, thus introducing an additional layer of complexity.

## The PRS situation today—uses and limitations

Currently, PRS assessments capture only a fraction of the total estimated heritable component of a trait [5, 6], partly because they are determined using only a limited number of polymorphic variants in certain genes. The PRSs are commonly calculated as a weighted sum of the number of disease risk (increasing/decreasing) variants carried by an individual, where the risk variants and their weighting, derived from genome-wide association studies (GWASs) [7, 8], may not be the relevant genetic factors but simply located nearby, thus introducing uncertainty in the estimates of effect size associated with individual variants in PRS. The GWASs are typically carried out in populations of defined ancestry (commonly European) and the data extrapolated from those studies might not be valid for populations of different ancestries. As such their general applicability can also be limited.

Importantly, individual variants may increase the risk for one trait, while simultaneously reducing the risk of another. This complexity is often not obvious to individuals who request information about their future risk through PRS, because they are only informed about the risk for a specific trait that they have sought advice for. They are therefore not provided with data about the risks or benefits of another trait influenced by the same variants, which may or may not be known and might also have included those with effects on prenatal development.

Given the many limitations summarised above, PRSs are not used in clinics. However, it seems plausible that, in the near future, some may be introduced into clinical assessment with the aim of improving the identification of at-risk individuals, and treatment for specific conditions [9, 10]. However, this would not necessarily be translated into implementation for prenatal diagnostics.

In a proper clinical or research setting, an assessment of all potential contributory risks, including genetic and environmental ones, would be undertaken and made available. Outside this framework, and especially when PRS assessments are provided as direct-to-consumer tests, their evaluation of a patient’s risk may be dangerously incomplete and can lead to grave misunderstandings [1, 11]. Extrapolating the results from predictive assessments in adult cohorts to use them as a factor for embryo screening would be improper. No clinical research protocol has been performed so far to assess the diagnostic effectiveness of PRSs in embryos. Were these be established, it would take many years to obtain reliable results, given that one might have to wait decades for people to develop, for example, early-onset Alzheimer’s disease.

## The use of PRS in embryo screening and selection

While it is relatively common for parents to consider any genetic risks they may pass on to their children, this is normally undertaken via the proven practice of carrier screening and genetic testing for inherited Mendelian disorders. In these cases, the ability of the test to predict the development of the disease is usually very high. In fact, when a genetic condition has an extremely low penetrance (the proportion of people with a particular genetic variant who exhibit signs and symptoms of a genetic disorder is low), it is very rare that the prospective parents would even consider prenatal or pre-implantation testing.

When applied to the selection of embryos for transfer, the PRS will relate to an individual family and not to a wide population. The intrafamilial variability would be much more limited than in the wider population, and therefore the PRS would be unlikely to be useful in determining the choice of one embryo over another, particularly as the number of viable embryos available is typically very small. Even if a discrete difference exists between two or more viable embryos suitable for transfer, a particular combination of genetic variants detected and evaluated would not provide a definitive diagnosis. Such a set of variants will correspond at best to a small increase in an individual’s risk, relative to the population’s risk for a complex trait, if the prediction is based on estimates for an ethnic group (ancestry) corresponding to that of the parents. In addition, if the selection were aimed at more than one PRS per embryo, it is easy to estimate by simple probability that the total number of embryos needed to be examined in order to find at least one (if any) suitable embryos to transfer would be unrealistic for our species and would also be unethical.

Overall, adding PRSs to PGT would amount to a form of embryo screening. The criteria to assess and implement a screening programme would include, among others, the proportionality principle, according to which “the possible benefits of the screening should clearly outweigh its possible disadvantages”. For the assessment of the proportionality of PRSs in PGT, it is important to take account of tensions with other parameters, more important for ranking embryos for transfer. Such parameters include viability scores and implications for the complex counselling process, especially when the values of professionals and customers for embryo ranking do not match.

Research on PRSs is not aimed at the development of pre-symptomatic tests in embryos but rather at the advancement of understanding of disease mechanisms, and the management and treatment of liveborn individuals, most frequently when they reach their adulthood. For PRS research, the aim is different, the population is different, the setting is different from what is expected from PGT.

## Protecting prospective parents, their offspring, and society

At present, carrying out a PRS test for embryo selection would be premature at best. Prospective parents and the public must be provided with adequate and unbiased information on the risks and limitations of such a practice [12]. It will be vital that a societal debate takes place before any potential application of the technique, and this should be focused on what would be considered acceptable with regard to the selection of individual traits, in particular. Without proper public engagement and oversight, the practice of implementing PRS test for embryo selection could easily lead to discrimination and the stigmatisation of certain conditions.

Further studies are needed to understand which and how polygenic risk estimates for common diseases can be implemented in clinical care. Such research should disentangle the complex interplay between PRSs for a range of conditions and the environment. More studies are needed to understand the biology of normal embryonic and foetal development, as well as its interplay with the intrauterine environment, which is still so elusive.

For the time being, it is important for reasons of justice to assess whether public and individual resources can be better used to improve our knowledge on PRSs and their relationships with the environment in which we live, rather than on the premature application of an inadequately evaluated test to our future children.

## References

[1] Turley P, Meyer MN, Wang N, Cesarini D, Hammonds E, Martin AR (2021). Problems with using polygenic scores to select embryos. N Engl J Med.

[2] Conley D. A new age of genetic screening is coming—and we don’t have any rules for it. The Washington Post; Washington, USA. 2021.

[3] Davis KW. A new kind of embryo genetics screening makes big promises on little evidence. Slate; Washington, USA. 2021.

[4] Goldberg C. Picking embryos with best health odds sparks new DNA debate. Bloomberg News; Washington, USA. 2021.

[5] Janssens ACJW, Joyner MJ (2019). Polygenic risk scores that predict common diseases using millions of single nucleotide polymorphisms: is more, better?. Clin Chem.

[6] Wald NJ, Old R (2019). The illusion of polygenic disease risk prediction. Genet Med.

[7] Martens FK, Tonk ECM, Jansens ACJW (2019). Evaluation of polygenic risk models using multiple performance measures: a critical assessment of discordant results. Genet Med.

[8] Wand H, Lambert SA, Tamburro C, Iacocca MA, O’Sullivan JW, Sillari C, et al. Improving reporting standards for polygenic scores in risk prediction studies. Nature. 2021;591:211–9 10.1038/s41586-021-03243-6 PMC8609771 33692554

[9] Lewis CM, Vassos E (2020). Polygenic risk scores: from research tools to clinical instruments. Genome Med.

[10] Moorthie S, Babb de Villiers C, Brigden T, Gaynor L, Hall A, Johnson E, et al. Polygenic scores, risk and cardiovascular disease. PHG Foundation, Cambridge, UK. 2019. www.phgfoundation.org.

[11] Horton R, Crawford G, Freeman L, Fenwick A, Wright CF, Lucassen A (2019). Direct-to-consumer genetic testing. BMJ..

[12] Pagnaer T, Siermann M, Borry P, Tšuiko O (2021). Polygenic risk scoring of human embryos: a qualitative study of media coverage. BMC Med Ethics.

