# Correction: The use of polygenic risk scores in pre-implantation genetic testing: an unproven, unethical practice

**DOI:** 10.1038/s41431-022-01155-1

**Published:** 2022-08-19

**Authors:** Francesca Forzano, Olga Antonova, Angus Clarke, Guido de Wert, Sabine Hentze, Yalda Jamshidi, Yves Moreau, Markus Perola, Inga Prokopenko, Andrew Read, Alexandre Reymond, Vigdis Stefansdottir, Carla van El, Maurizio Genuardi, on behalf of the Executive Committee of the European Society of Human Genetics, Borut Peterlin, Carla Oliveira, Karin Writzl, Gunnar Douzgos Houge, the Public and Professional Policy Committee of the European Society of Human Genetics, Christophe Cordier, Heidi Howard, Milan Macek, Béla Melegh, Alvaro Mendes, Dragica Radojkovic, Emmanuelle Rial-Sebbag, Fiona Ulph

**Affiliations:** 1 Clinical Genetics Department, Guy’s and St Thomas NHS Foundation Trust, London, UK; 2 Department of Medical Genetics, Medical University of Sofia, Sofia, Bulgaria; 3 Division of Cancer and Genetics, School of Medicine, Cardiff University, Cardiff, Wales UK; 4 Maastricht University, Maastricht, The Netherlands; 5 Human Genetics, Heidelberg, Germany; 6 Genetics Research Centre, Molecular and Clinical Sciences Institute, St George’s University of London, London, UK; 7 University of Leuven ESAT-STADIUS, B-3001 Leuven, Belgium; 8 Finnish Institute for Health and Welfare (THL), Biomedicum 1, Haartmaninkatu 8, 00290 Helsinki, Finland; 9 Department of Clinical & Experimental Medicine, University of Surrey, Guildford, UK; 10 UMR 8199 - EGID, Institut Pasteur de Lille, CNRS, University of Lille, F-59000 Lille, France; 11 Institute of Biochemistry and Genetics, Ufa Federal Research Centre, Russian Academy of Sciences, Ufa, Russian Federation; 12 Centre for Genomic Medicine, St Mary’s Hospital, Manchester, M13 0JH England; 13 Center for Integrative Genomics, University of Lausanne, CH-1015 Lausanne, Switzerland; 14 Department of Genetics and Molecular Medicine, Landspitali University Hospital, Reykjavik, Iceland; 15 Section Community Genetics, Department of Clinical Genetics and Amsterdam Public Health Research Institute, Amsterdam UMC, Vrije Universiteit Amsterdam, Amsterdam, The Netherlands; 16 UOC Genetica Medica, Dipartimento di Scienze di Laboratorio e Infettivologiche, Fondazione Policlinico Universitario A. Gemelli IRCCS, Rome, Italy; 17 Sezione di Medicina Genomica, Dipartimento di Scienze della Vita e Sanità Pubblica, Università Cattolica del Sacro Cuore, Rome, Italy; 18 Clinical Institute for Genomic Medicine, University Medical Center Ljubljana, Ljubljana, Slovenia; 19 Department of Pathology, Faculty of Medicine, University of Porto, PT, Porto, Portugal; 20 Instituto de Investigação e Inovação em Saúde (i3S), University of Porto, PT, Porto, Portugal; 21 Institute of Molecular Pathology and Immunology of the University of Porto (Ipatimup), University of Porto, PT, Porto, Portugal; 22 Department of Medical Genetics, Haukeland University Hospital, 5021 Bergen, Norway; 23 Department of Genetics, SYNLAB Suisse SA, Chemin d’Entre Bois 21, 1018 Lausanne, Switzerland; 24 Researcher Medical Ethics, Lund University, Uppsala, Sweden; 25 Chalmers University (part of GENIE initiative), Uppsala, Sweden; 26 Department of Biology and Medical Genetics, 2nd Faculty of Medicine, Charles University and Motol University Hospital, V Uvalu 84, Prague, CZ15006 Czech Republic; 27 Department of Medical Genetics, University of Pécs, Szigeti 12., H-7624 Pécs, Hungary; 28 UnIGENe and Centre for Predictive and Preventive Genetics, IBMC—Institute for Molecular and Cell Biology, i3S—Instituto de Investigação e Inovação em Saúde, Universidade do Porto, Porto, Portugal; 29 Laboratory for Molecular Biology, Institute of Molecular Genetics and Genetic Engineering, University of Belgrade, Belgrade, Serbia; 30 CERPOP, UMR 1295, Inserm, CERPOP, UMR 1295, Inserm, Université de Toulouse-Université Paul Sabatier-Toulouse III, Responsable Equipe BIOETHICS: Trajectoires d’innovations en santé:enjeux bioéthiques et sociétaux, Toulouse, France; 31 Plateforme Sociétale “Génétique et Société, GIS Genotoul, Génopole Toulouse Midi-Pyrénées, 37, allées Jules Guesde, 31073 Toulouse Cedex, France; 32 Manchester Centre of Health Psychology, Division of Psychology and Mental Health, School of Health Sciences, Manchester Academic Health Science Centre, University of Manchester, Coupland Street, Manchester, M13 9PL England

**Keywords:** Medical ethics, Disease prevention

Correction to: *European Journal of Human Genetics* 2021;30:493–5 10.1038/s41431-021-01000-x

The article “The use of polygenic risk scores in pre-implantation genetic testing: an unproven, unethical practice”, written by Francesca Forzano et al., was originally published electronically on the publisher’s internet portal on 17 December 2021 without open access. With the authors’ decision to opt for Open Choice, the copyright of the article changed on 11 July 2022 to © Author(s) 2021 and the article is forthwith distributed under a Creative Commons Attribution 4.0 International License, which permits use, sharing, adaptation, distribution and reproduction in any medium or format, as long as you give appropriate credit to the original author(s) and the source, provide a link to the Creative Commons licence and indicate if changes were made. The images or other third-party material in this article are included in the article’s Creative Commons licence, unless indicated otherwise in a credit line to the material. If material is not included in the article’s Creative Commons licence and your intended use is not permitted by statutory regulation or exceeds the permitted use, you will need to obtain permission directly from the copyright holder. To view a copy of this licence, visit http://creativecommons.org/licenses/by/4.0.

The original article has been corrected.

